# *E. coli*-expressed SECRET AGENT *O*-GlcNAc modifies threonine 829 of GIGANTEA

**DOI:** 10.3389/fpls.2024.1343066

**Published:** 2024-07-18

**Authors:** Young-Cheon Kim, Lynn M. Hartweck, Neil E. Olszewski

**Affiliations:** The Department of Plant and Microbial Biology, University of Minnesota, St. Paul, MN, United States

**Keywords:** GIGANTEA, O-GlcNAc, SECRET AGENT, posttranslational modification, mass spectrometry

## Abstract

The *Arabidopsis thaliana* glycosyl transferases SPINDLY (SPY) and SECRET AGENT (SEC) modify nuclear and cytosolic proteins with *O*-linked fucose or *O*-linked *N*-acetylglucosamine (*O*-GlcNAc), respectively. *O*-fucose and *O*-GlcNAc modifications can occur at the same sites. SPY interacts physically and genetically with GIGANTEA (GI), suggesting that it could be modified by both enzymes. Previously, we found that, when co-expressed in *Escherichia coli*, SEC modifies GI; however, the modification site was not determined. By analyzing the overlapping sub-fragments of GI, we identified a region that was modified by SEC in *E. coli*. Modification was undetectable when threonine 829 (T829) was mutated to alanine, while the T834A and T837A mutations reduced the modification, suggesting that T829 was the primary or the only modification site. Mapping using mass spectrometry detected only the modification of T829. Previous studies have shown that the positions modified by SEC in *E. coli* are modified *in planta*, suggesting that T829 is *O*-GlcNAc modified *in planta*.

## 1 Introduction

The posttranslational modification of serine or threonine residues with *O*-linked β-*N*-acetylglucosamine (*O*-GlcNAc) regulates the activity of nuclear and cytosolic proteins (Ma et al., 2022). *Arabidopsis thaliana* has one *O*-GlcNAc transferase, SECRET AGENT (SEC) (Hartweck et al., 2002). *Arabidopsis* also has an *O*-fucose transferase, SPINDLY (SPY), which also modifies the serine and threonine of nuclear and cytosolic proteins (Zentella et al., 2017). *sec* mutants have subtle defects, while *spy* mutants have stronger defects (Hartweck et al., 2002, Hartweck et al., 2006; Zentella et al., 2016; Sun, 2021). *sec spy* double mutants are embryo-lethal, suggesting that SEC and SPY have overlapping functions (Hartweck et al., 2002). Recent surveys have identified a number of *O*-GlcNAc- and *O*-fucose-modified plant proteins (Xu et al., 2017; Wu et al., 2022; Bi et al., 2023; Li et al., 2023; Zentella et al., 2023). Consistent with SEC and SPY having overlapping functions, *O*-GlcNAc and *O*-fucose modifications can occur at the same position (Xu et al., 2017; Bi et al., 2023).

GIGANTEA (GI), a protein that regulates many processes including the circadian clock, flowering time, and light signaling (Mishra and Panigrahi, 2015; Krahmer et al., 2019; Brandoli et al., 2020), interacts physically and genetically with SPY (Tseng et al., 2004). Previously, we found that, when co-expressed in *Escherichia coli*, SEC modifies GI, but the location of the modification was not determined (Kim et al., 2013). In this study, we used deletion analysis, site-directed mutagenesis, and mass spectrometry (MS) to map the location of the modification of GI by SEC.

## 2 Materials and methods

### 2.1 GI expression constructs

To create the *E. coli* expression constructs, portions of the GI protein coding region were amplified by PCR using the primers listed in **Supplementary Table S1** and cloned between the *Bam*HI and *Not*I sites of pET32a. This region of GI was produced with N-terminal S- and His-tags. To create the pET32-CT5, the region encoding GI amino acids 789–893 (GenBank: AAT80910.1) (**Supplementary Figure S1**) was cloned between the *Nco*I and *Xho*I sites of pET32a. The protein expressed from this construct was named CT5. Site-directed mutagenesis of pET32-CT5 was performed using the QuikChange Site-Directed Mutagenesis Kit following the instructions of the manufacturer (Stratagene, La Jolla, CA, USA) using the primers listed in **Supplementary Table S1**.

### 2.2 Detection of GlcNAcylated proteins on the protein blots

SEC and different portions of GI were co-expressed for 2 h in BL21-AI essentially as described previously (Scott et al., 2006). Cells were harvested from 1.5 mL of culture by centrifugation and resuspended in 50 µL of phosphate-buffered saline (PBS) and 17 µL of 4× sodium dodecyl sulfate (SDS) gel-loading buffer. The cells were lysed by boiling for 5 min, and the sample proteins were resolved using SDS-PAGE, blotted to Immobilon-P membrane (Millipore, Bedford, MA, USA), and the blots probed to detect the GlcNAc-modified proteins using a radioactive galactosyl transferase assay that capped GlcNAc with ^3^H-galactose (Scott et al., 2006). To detect labeled proteins, the membrane was sprayed with EN^3^HANCE (Perkin-Elmer Life Science, Boston, MA, USA), air-dried, and exposed to a pre-flashed BioMax XAR film (Eastman Kodak, Rochester, NY, USA) at −80°C. S-tagged proteins were detected on duplicate blots using horseradish peroxidase-conjugated anti-S antibodies (Novagen, Madison, WI, USA) as recommended by the supplier. Horseradish peroxidase was detected using Super Signal West Pico (Pierce, Rockford, IL, USA) with exposure to X-OMAT™ Blue XB-1 film (Eastman Kodak, Rochester, NY, USA).

### 2.3 Enrichment of *O*-GlcNAc-modified peptides using RCA I lectin

The purified *O*-GlcNAc-modified CT5 was prepared as described previously (Kim et al., 2011). SEC and CT5 were co-expressed from pET32-CT5 and pACYC-Mal-SEC in *E. coli* BL21-AI. Of a Luria–Bertani (LB) medium, 500 mL was inoculated with 10 mL of an overnight culture and grown at 22°C. Arabinose was added to 0.2% (*w*/*v*) when the culture reached an optical density (OD_600_) of 0.4. After 1 h, isopropyl-β-d-thiogalactoside was added to 1 mM and the cells harvested after 2 h by centrifugation. The cells were resuspended in 20 mL of 50 mM sodium phosphate and 500 mM sodium chloride (pH 8.0) and broken using a French press (Milton Roy, Ivyland, PA, USA) at 10,000 psi. CT5 was purified using the ProBond Purification System (Invitrogen Life Technologies, Carlsbad, CA, USA) following the manufacturer’s instructions. The purified proteins were concentrated using an Amicon Ultra-15 (Millipore Corporation, Billerica, MA, USA). The purified CT5 was then reduced in 2 mM Tris-(2-carboxyethyl)phosphine at room temperature for 1 h and alkylated in 10 mM iodoacetamide in the dark at room temperature for 1.5 h. The alkylated CT5 was digested with MS grade trypsin (40:1; Promega, Madison, WI, USA) or endoproteinase Lys-C (100:1; Roche, Penzberg, Germany) overnight at 37°C, desalted using a Sep-Pak C_18_ cartridge (Waters, Milford, MA, USA), and then dried using a Speed-Vac. GlcNAc was capped with galactose using galactosyl transferase, creating the disaccharide *N*-acetyl-d-lactosamine (LacNAc). Peptides bearing LacNAc were enriched by RCA I affinity chromatography (Hayes et al., 1995; Haynes and Aebersold, 2000). The peptides were dissolved in 100 μL PBS and loaded onto a 1.2-m-long RCA I agarose column (Vector Laboratories, Burlingame, CA, USA) constructed in a Teflon tubing (1.55 mm i.d. × 1.2 m length, with a 0.5-μm end frit). The column was washed at room temperature with 3 mL PBS at a flow rate of 50 uL/min and 100 uL fractions were collected. The bound material was then eluted with 0.2 M lactose in PBS. Peptides were detected by measuring the absorbance at 280 nm. The pool was dried in a Speed-Vac, then desalted using OMIS C_18_/100 μL (Varian, Palo Alto, CA, USA), and dried again (Kim et al., 2011).

### 2.4 Removal of *O*-linked LacNAc by β-elimination

For β-elimination, the desalted and dried peptides were resuspended in 1.5% triethylamine and 0.15% NaOH and then incubated at 52°C for 1.5 h. The peptides were then desalted using Sep-Pak C_18_, dried, and stored at −20°C.

### 2.5 Matrix-assisted laser desorption ionization time-of-flight analysis

The peptide samples were purified with a C_18_ ZipTip (Millipore, Bedford, MA, USA). Approximately 1.2 µL of the eluted peptides was mixed on the target with the matrix (10 mg/mL α-cyano-4-hydroxycinnamic acid in 50% acetonitrile and 0.1% trifluoroacetic acid) and analyzed in reflector mode on a matrix-assisted laser desorption ionization time-of-flight (MALDI-TOF) MS using Bruker Reflex III (Bruker Daltonics, Billerica, MA, USA). Processing of the spectra and data analysis were performed with the Bruker Daltonics XTOF 3.1.

### 2.6 Nanoflow LC-MS/MS and data analysis

Online reversed-phase nanoflow HPLC with electrospray ionization MS, which used an LTQ-OrbitrapXL equipped with electron transfer dissociation (ETD) or collision-induced dissociation (CID) sources (Thermo Scientific, Waltham, MA, USA), and analysis of the MS data were performed as described previously (Bandhakavi et al., 2009; Kim et al., 2013). The peptides were eluted using an acetonitrile gradient of 2%–40% over 60 min. Survey scans from 360 to 1,800 *m*/*z* (mass-to-charge) were acquired using the Orbitrap analyzer at 60,000 resolution. The data-dependent settings included selection of the top five most abundant ions in each survey scan for tandem mass spectrometry (MS/MS), excluding 1+ or undetermined charge states; dynamic exclusion was enabled for 20 s. Fragment ions were detected in the linear ion trap for both CID and ETD activation modes. For experiments using ETD fragmentation, the scan parameters were as follows: precursor ion isolation window of 3 *m*/*z* units, precursor ion automatic gain control of 2 × 10^4^ charges, precursor injection time of 100 ms, fluoranthene reagent ion, reagent ion automatic gain control of 400,000, reagent ion injection time of 50 ms, and reagent ion reaction time of 100 ms. Fragmentation with CID was performed as described previously (Bandhakavi et al., 2009) with 35% normalized collision energy. The CID and ETD data were analyzed using Scaffold version 3 (www.proteomesoftware.com) and OMSSA version 2.1.7, respectively, and confirmed via manual inspection.

Prior to database searching, raw CID data were extracted and converted to the mzXML format using the MS Convert software from ProteoWizard. Data were searched using SEQUEST version 27, revision 12, against the National Center for Biotechnology Information (NCBI)-derived reference sequence *Arabidopsis* database from September 2009, which included common contaminants from http://www.thegpm.org/crap/index.htm and CT5. The precursor and fragment ion tolerances for the database searches were set at 10 ppm and 0.8 amu. Semi-tryptic specificity was selected with up to two missed cleavage sites. Since the samples had been alkylated, the addition of 57.02146 amu to Cys was set as a fixed modification, and variable modifications included the addition of 15.9949 amu to Met, to allow for its oxidation, and loss of 18.0106 amu from either serine or threonine, which would occur when the modification of lactosamine is removed by β-elimination. The probabilities of the peptide candidate identifications being correct (Keller et al., 2002) were calculated using Scaffold version 3. Protein identifications were filtered using the following criteria: 10 ppm precursor mass tolerance, more than 95% peptide probability, and full trypsin specificity prior to confirmation by manual inspection.

When the ETD data were analyzed using OMSSA,.dta files were generated using the DTA generator (https://github.com/coongroup/Compass?search=1). The MS/MS spectra were searched against all *Arabidopsis* proteins in the NCBI non-redundant database and CT5. The parameters for the search were the same as those described above, with the addition of allowing variable modification of 365.1322 amu to serine (S) and threonine (T).

## 3 Results

### 3.1 Mapping the GI modification by SEC using deletion analysis

**Figure 1:**
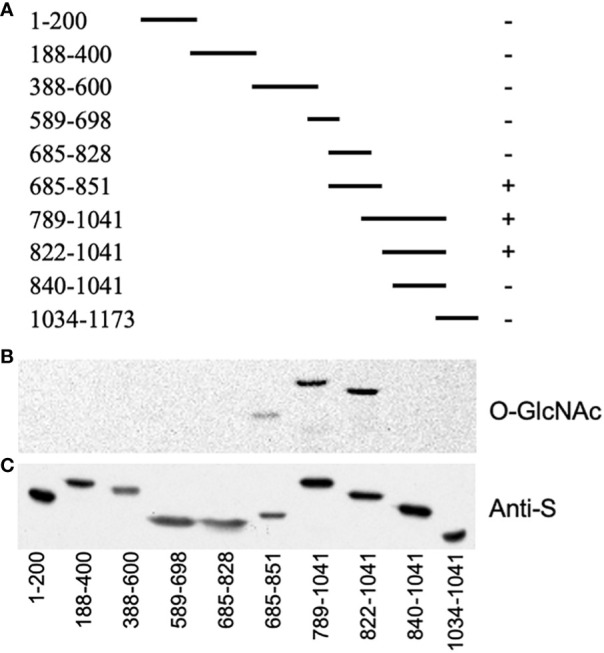
SECRET AGENT (SEC) modifies the region encompassing amino acids 828–840 of GIGANTEA (GI). **(A)** Map of the segments of GI that were co-expressed with SEC. The *plus sign* indicates that the protein was modified, while a *minus sign* indicates it was not. **(B)** GlcNAc modification of blotted proteins was detected using a galactosyl transferase assay (*O*-GlcNAc). **(C)** Duplicate blot probed with an anti-S antibody to confirm the expression of GI (anti-S).

As we were unable to co-express the full-length GI protein with SEC in *E. coli*, smaller segments that collectively span GI were co-expressed and analyzed for *O*-GlcNAc modification (**Figure 1A**). All fragments containing amino acids 828–840 were modified, suggesting that this region had been modified (**Figures 1B, C**).

**Figure 2:**
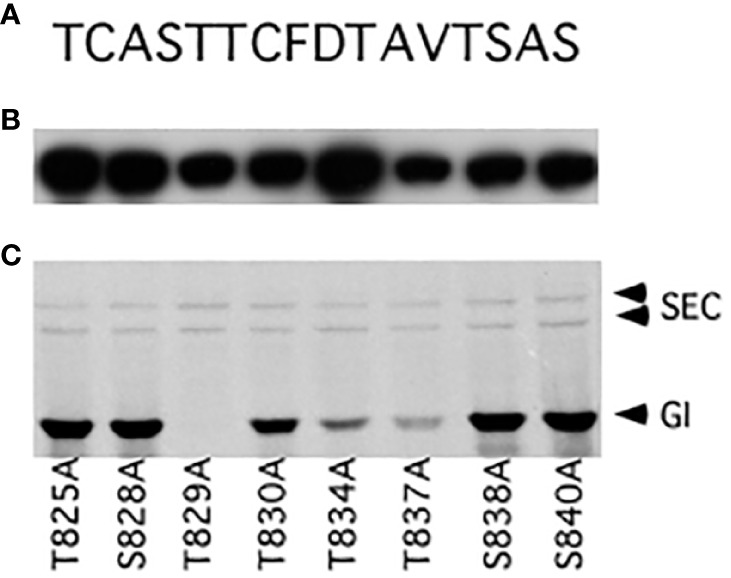
Mutational mapping of GIGANTEA (GI) *O*-GlcNAc modification sites. **(A)** Amino acid sequence of the GI 825–840 region. **(B)** Western blot using an anti-S antibody. **(C)** Fluorograph detecting *O*-GlcNAc modification of mutant GI proteins.

An *E. coli* expression construct termed pET32-CT5, which encodes GI amino acids 789–893, was expressed well, and the protein it encodes, named CT5, was highly modified when co-expressed with SEC (**Supplementary Figure S2A**). Therefore, this construct was used for further mapping studies. The serine (S) and threonine (T) residues of the region spanning amino acids 825–840 were individually mutated to alanine (A), and the mutant proteins were examined to determine whether they were modified by SEC (**Figure 2**). The T834A and T837A mutations reduced the modification, while modification of the T829A mutant was not detectable. Since the mutations might affect the protein structure or the interaction with SEC, or create an ectopic modification site, MS mapping was employed to unambiguously map the modification site(s).

### 3.2 Mass spectrometry demonstrates that T829 is *O*-GlcNAc modified

**Figure 3:**
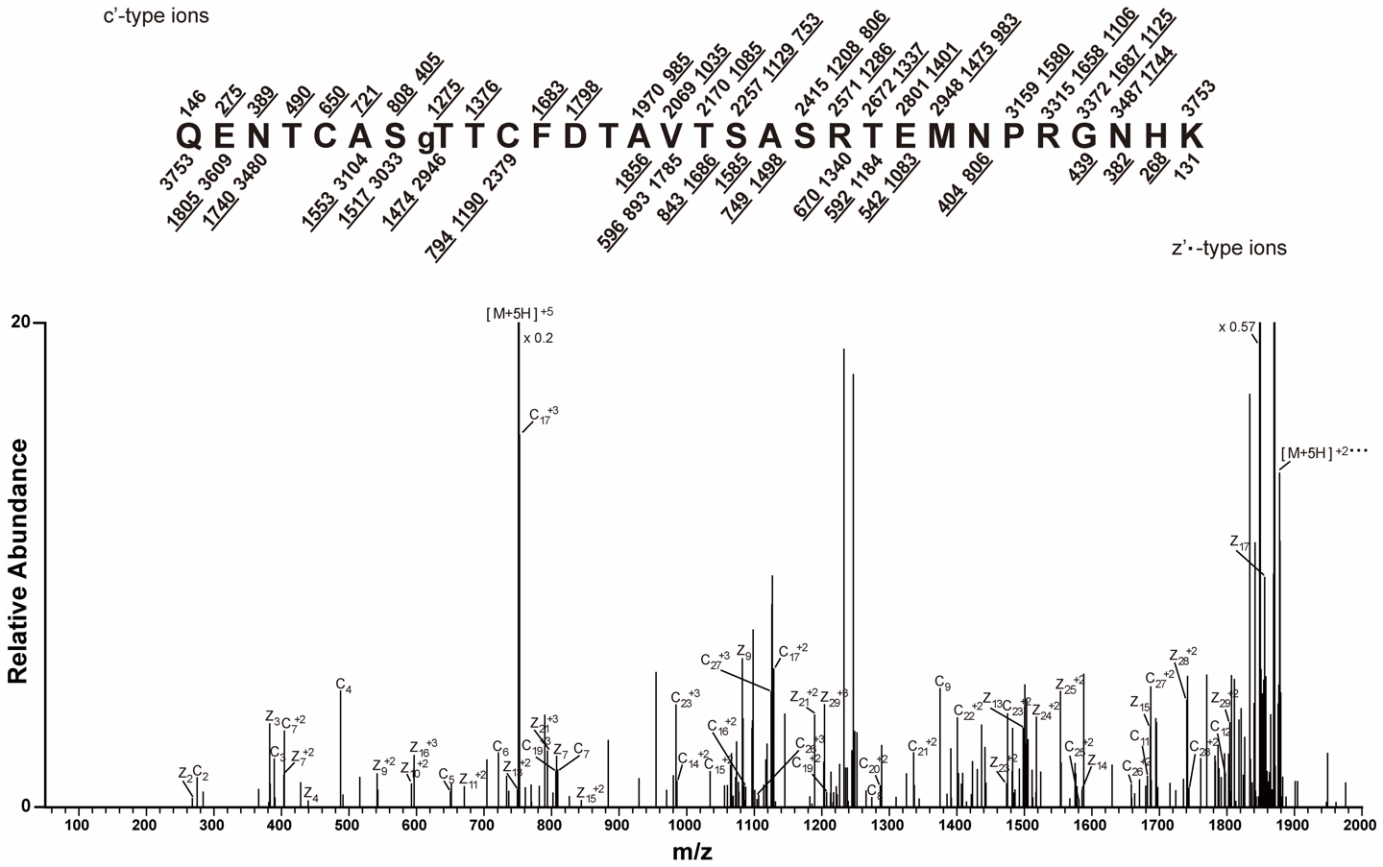
Mapping of the CT5 modification site by mass spectrometry. CT5 was digested with Lys-C, GlcNAc was capped with galactose, and modified peptides were enriched by RCA I affinity chromatography (**Supplementary Figure S2**). The electron transfer dissociation (ETD) tandem mass spectrometry (MS/MS) spectrum recorded on [M+5H]^+5^ ions (*m/z* 751.3275) from LacNAc (365.1322) modified the CT5 peptide QENTCASTgTCFDTAVTSASRTEMNPRGNHK. The predicted c′ and z′•-type ions are listed *above* and *below* the peptide sequence, respectively. Singly and doubly charged fragment ions are listed as monoisotopic masses. The ions observed and labeled in the spectrum are *underlined*. The residue at T829 is preceded by “g” to signify modification by a single LacNAc moiety.

The *O*-GlcNAc-modified CT5 peptides produced by digestion with Lys-C or trypsin were enriched by RCA I lectin affinity chromatography. MALDI-TOF analysis of the enriched peptides demonstrated successful enrichment of the modified peptides produced with either trypsin (*m/z* 2,573) or Lys-C digestion (*m/z* 3,736) (**Supplementary Figure S2**). When the RCA I-enriched Lys-C peptides were analyzed by ETD MS, a quintuple-charged precursor ion (*m*/*z* 751.3275) with the sequence QENTCASTTCFDTAVTSASRTEMNPRGNHK was observed to have c_8_ and z_23_ ions with a 365-Da (LacNAc) mass increase, which supports the *O*-GlcNAc modification of T829 (**Figure 3**).

**Figure 4:**
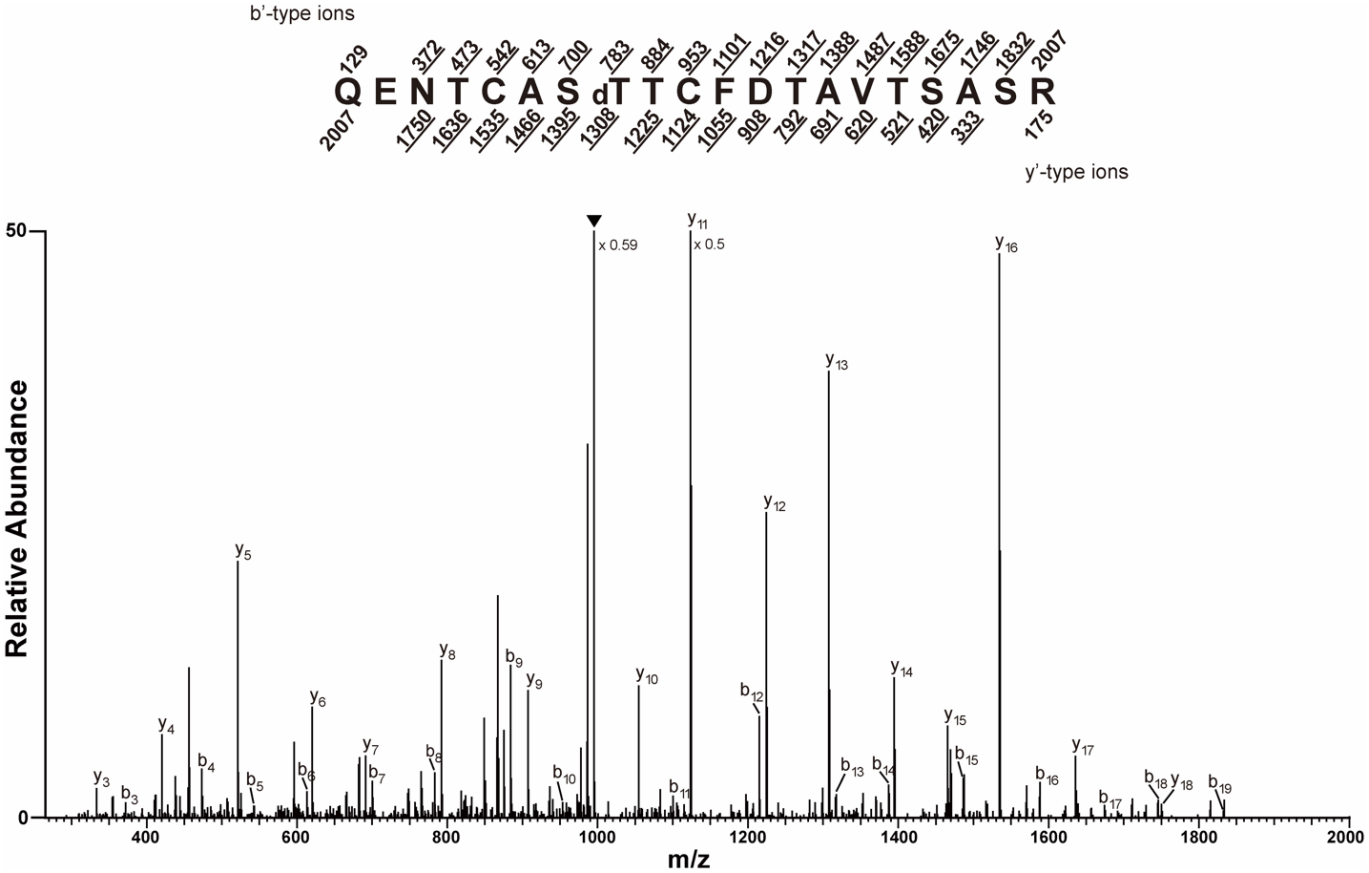
Trypsinized CT5 peptides were prepared, subjected to β-elimination, and the collision-induced dissociation (CID) tandem mass spectrometry (MS/MS) spectrum recorded on [M+2H]^+2^ ions (*m/z* 1,003.9561 and 1,003.9565) corresponding to the peptide QENTCASTdTCFDTAVTSASR dehydrated at the amino acid corresponding to GIGANTEA (GI) position T829. The predicted b′ and y′-type ions are listed *above* and *below* the peptide sequence, respectively. Singly charged fragment ions are listed as monoisotopic masses. The ions observed and labeled in the spectrum are *underlined*. The modified residue is preceded by “d” to signify the dehydrated T. The *filled upside down triangle* indicates [M+2H–H_2_O]^+2^ ions.

To confirm that the modification was *O*-linked, the RCA I-enriched trypsin peptides were subjected to β-elimination, which removed the *O*-linked modifications, and analyzed by CID MS. This analysis detected a double-charged precursor ion (*m*/*z* 1,003.9561) corresponding to the dehydrated QENTCASTTCFDTAVTSASR peptide, which is consistent with the removal of an *O*-linked modification. The b_8_ and y_13_ ion masses indicated that T829 was dehydrated (**Figure 4**).

## 4 Discussion

When co-expressed with SEC in *E. coli*, GI is *O*-GlcNAc modified. Through a combination of deletion, mutation, and MS analyses, it was shown that a single amino acid, i.e., T829, was modified. Our results suggest that only this site was modified. Several proteins including the RGA and TCP proteins and the coat protein of the plum pox virus are modified by SEC in plants and in *E. coli* (Scott et al., 2006; Kim et al., 2011; Steiner et al., 2012; Zentella et al., 2016; Xu et al., 2017). Five positions on the plum pox coat protein are modified by SEC in *E. coli*, and all of these positions are modified on virions isolated from *Nicotiana clevelandii* plants (Perez Jde et al., 2013). Thus, the substrate specificities of SEC in *E. coli* and *in planta* are similar, suggesting that the T829 of GI is *O*-GlcNAc modified in plants. However, the *O*-GlcNAc modification of GI was not identified in recent global analyses of *O*-GlcNacylation (Xu et al., 2017; Wu et al., 2022; Li et al., 2023). The protein abundance of GI is subject to circadian fluctuations, being most abundant in the evening (Krahmer et al., 2019), and thus could have been missed in these global analyses.

Studies identifying *O*-fucose- and *O*-GlcNAc-modified proteins have found that both modifications can occur at the same position (Bi et al., 2023; Zentella et al., 2023). Since GI interacts physically and genetically with the *Arabidopsis O*-fucose transferase SPY (Tseng et al., 2004; Zentella et al., 2017), it would be interesting to find out whether SPY also modifies GI at T829 and how *O*-glycosylation affects GI function.

## Data availability statement

The original contributions presented in the study are publicly available. This data can be found here: ProteomeXchange, PXD053909.

## Author contributions

Y-CK: Writing – review & editing, Writing – original draft, Investigation, Conceptualization. LH: Writing – review & editing, Writing – original draft, Investigation, Conceptualization. NO: Writing – review & editing, Writing – original draft, Funding acquisition, Conceptualization.

## References

[B1] Bandhakavi S. Stone M. D. Onsongo G. Van Riper S. K. Griffin T. J. (2009). A dynamic range compression and three-dimensional peptide fractionation analysis platform expands proteome coverage and the diagnostic potential of whole saliva. J. Proteome Res. 8, 5590–5600. doi: 10.1021/pr900675w 19813771 PMC2789208

[B2] Bi Y. Shrestha R. Zhang Z. Hsu C. C. Reyes A. V. Karunadasa S.. (2023). SPINDLY mediates O-fucosylation of hundreds of proteins and sugar-dependent growth in Arabidopsis. Plant Cell. 35, 1318–1333. doi: 10.1093/plcell/koad023 PMC10118272 36739885

[B3] Brandoli C. Petri C. Egea-Cortines M. Weiss J. (2020). Gigantea: uncovering new functions in flower development. Genes (Basel) 11, 1142. doi: 10.3390/genes11101142 32998354 PMC7600796

[B4] Hartweck L. M. Genger R. K. Grey W. M. Olszewski N. E. (2006). SECRET AGENT and SPINDLY have overlapping roles in the development of Arabidopsis thaliana L. Heyn. J. Exp. Bot. 57, 865–875. doi: 10.1093/jxb/erj071 16473894

[B5] Hartweck L. M. Scott C. L. Olszewski N. E. (2002). Two O-linked N-acetylglucosamine transferase genes of Arabidopsis thaliana L. Heynh. Have overlapping functions necessary for gamete and seed development. Genetics 161, 1279–1291. doi: 10.1093/genetics/161.3.1279 12136030 PMC1462182

[B6] Hayes B. K. Greis K. D. Hart G. W. (1995). Specific isolation of O-linked N-acetylglucosamine glycopeptides from complex mixtures. Analytical Biochem. 228, 115–122. doi: 10.1006/abio.1995.1322 8572267

[B7] Haynes P. A. Aebersold R. (2000). Simultaneous detection and identification of O-GlcNAc-modified glycoproteins using liquid chromatography-tandem mass spectrometry. Anal. Chem. 72, 5402–5410. doi: 10.1021/ac000512w 11080893

[B8] Keller A. Nesvizhskii A. I. Kolker E. Aebersold R. (2002). Empirical statistical model to estimate the accuracy of peptide identifications made by MS/MS and database search. Anal Chem. 74, 5383–5392. 12403597 10.1021/ac025747h

[B9] Kim Y. C. Jahren N. Stone M. D. Udeshi N. D. Markowski T. W. Witthuhn B. A.. (2013). Identification and origin of N-linked beta-D-N-acetylglucosamine monosaccharide modifications on Arabidopsis proteins. Plant Physiol. 161, 455–464. doi: 10.1104/pp.112.208900 23144189 PMC3532274

[B10] Kim Y. C. Udeshi N. D. Balsbaugh J. L. Shabanowitz J. Hunt D. F. Olszewski N. E. (2011). O-GlcNAcylation of the Plum pox virus capsid protein catalyzed by SECRET AGENT: characterization of O-GlcNAc sites by electron transfer dissociation mass spectrometry. Amino Acids 40, 869–876. doi: 10.1007/s00726-010-0706-0 20676902 PMC3159405

[B11] Krahmer J. Goralogia G. S. Kubota A. Zardilis A. Johnson R. S. Song Y. H.. (2019). Time-resolved interaction proteomics of the GIGANTEA protein under diurnal cycles in Arabidopsis. FEBS Lett. 593, 319–338. doi: 10.1002/1873-3468.13311 30536871 PMC6373471

[B12] Li X. L. Lei C. Song Q. T. Bai L. Cheng B. Qin K.. (2023). Chemoproteomic profiling of O-GlcNAcylated proteins and identification of O-GlcNAc transferases in rice. Plant Biotechnol. J. 21, 742–753. doi: 10.1111/pbi.13991 36577688 PMC10037131

[B13] Ma J. Hou C. Wu C. (2022). Demystifying the O-glcNAc code: A systems view. Chem. Rev. 122, 15822–15864. doi: 10.1021/acs.chemrev.1c01006 35302357

[B14] Mishra P. Panigrahi K. C. (2015). GIGANTEA - an emerging story. Front. Plant Sci. 6. doi: 10.3389/fpls.2015.00008 PMC4306306 25674098

[B15] Perez Jde J. Udeshi N. D. Shabanowitz J. Ciordia S. Juarez S. Scott C. L.. (2013). O-GlcNAc modification of the coat protein of the potyvirus Plum pox virus enhances viral infection. Virology 442, 122–131. doi: 10.1016/j.virol.2013.03.029 23639873 PMC4625898

[B16] Scott C. L. Hartweck L. M. de Jesus Perez J. Chen D. Garcia J. A. Olszewski N. E. (2006). SECRET AGENT, an Arabidopsis thaliana O-GlcNAc transferase, modifies the Plum pox virus capsid protein. FEBS Lett. 580, 5829–5835. doi: 10.1016/j.febslet.2006.09.046 17027982

[B17] Steiner E. Efroni I. Gopalraj M. Saathoff K. Tseng T. S. Kieffer M.. (2012). The Arabidopsis O-linked N-acetylglucosamine transferase SPINDLY interacts with class I TCPs to facilitate cytokinin responses in leaves and flowers. Plant Cell 24, 96–108. doi: 10.1105/tpc.111.093518 22267487 PMC3289577

[B18] Sun T. P. (2021). Novel nucleocytoplasmic protein O-fucosylation by SPINDLY regulates diverse developmental processes in plants. Curr. Opin. Struct. Biol. 68, 113–121. doi: 10.1016/j.sbi.2020.12.013 33476897 PMC8222059

[B19] Tseng T. S. Salome P. A. McClung C. R. Olszewski N. E. (2004). SPINDLY and GIGANTEA interact and act in Arabidopsis thaliana pathways involved in light responses, flowering, and rhythms in cotyledon movements. Plant Cell 16, 1550–1563. doi: 10.1105/tpc.019224 15155885 PMC490045

[B20] Wu J. Lei C. Li X. Dong X. Qin K. Hong W.. (2022). Chemoproteomic profiling of O-glcNAcylation in arabidopsis thaliana by using metabolic glycan labeling. Israel J. Chem. 63, e202200065. doi: 10.1002/ijch.202200065

[B21] Xu S. L. Chalkley R. J. Maynard J. C. Wang W. F. Ni W. M. Jiang X. Y.. (2017). Proteomic analysis reveals O-GlcNAc modification on proteins with key regulatory functions in Arabidopsis. Proc. Of Natl. Acad. Of Sci. Of United States Of America 114, E1536–E1543. doi: 10.1073/pnas.1610452114 PMC5338445 28154133

[B22] Zentella R. Hu J. Hsieh W. P. Matsumoto P. A. Dawdy A. Barnhill B.. (2016). O-GlcNAcylation of master growth repressor DELLA by SECRET AGENT modulates multiple signaling pathways in Arabidopsis. Genes Dev. 30, 164–176. doi: 10.1101/gad.270587.115 26773002 PMC4719307

[B23] Zentella R. Sui N. Barnhill B. Hsieh W. P. Hu J. H. Shabanowitz J.. (2017). The Arabidopsis O-fucosyltransferase SPINDLY activates nuclear growth repressor DELLA. Nat. Chem. Biol. 13, 479–47+. doi: 10.1038/nchembio.2320 28244988 PMC5391292

[B24] Zentella R. Wang Y. Zahn E. Hu J. Jiang L. Shabanowitz J.. (2023). SPINDLY O-fucosylates nuclear and cytoplasmic proteins involved in diverse cellular processes in plants. Plant Physiol. 191, 1546–1560. doi: 10.1093/plphys/kiad011 36740243 PMC10022643

