# Supplementary material for: E. coli-expressed SECRET AGENT O-GlcNAc modifies threonine 829 of GIGANTEA
Source: Front Plant Sci. 2024 Jul 18;15:1343066. doi: 10.3389/fpls.2024.1343066 (PMC11291313; doi:10.3389/fpls.2024.1343066)
Supplement: Supplementary file 1 [file DataSheet_1.docx]

**Supplemental Table 1** Testing of GI modification across different regions of GI. The amino acids encoded by the amplified region or the CT5 amino acid mutations are listed in the first column. The forward and reverse primers used to make the GI fragments by PCR amplification are listed. The results of testing whether SEC modified the fragments (shown in Figure 1) are summarized in the last column.

| Region expressed/mutated | Primers | O-GlcNac modified by SEC |
| --- | --- | --- |
| 1-200 | Lynn/GiBam Atg32a  Cggatccatggctagttcatcttcatctga  Gi600F  cagcggccgctaAGCAAGTAGTATATCACTGAT | No |
| 188-400 | Gi562F  cggatccAGGCCTTTGTCTCCATGGATC  Gi1200R  cagcggccgctaCGGCTGTGAGAGTATGCGGAA | No |
| 388-600 | Gi1162F  CggatccGCTGCAGCCGCTTTGCTTTTC  Gi1800R  cagcggccgctaTTCATGGCTAACACATACAGT | No |
| 589-698 | Lynn/Gi1765F  cggatccCTTTTTGTTGTGTTGACTG  Lynn/Gi2093R  cagcggccgctaTCCATACTCTTTAGATGACCC | No |
| 685-828 | Lynn/Gi2053F  cggatccACAAAGCCTGTAAAGATAAATGGG  Gi2464R  cagcggccgctaGCTTGCACATGTGTTCTCTTG | No |
| 685-851 | Lynn/Gi2053F  cggatccACAAAGCCTGTAAAGATAAATGGG  Gi2551R  cagcggccgctaCTTATGGTTTCCTCTTGGATT | Yes |
| 789-1041 | Gi2365F  cggatccAAAGTTGTTGCCTCCATTGTTGAC  Gi3123R  cagcggccgctaGGCTTCAAGTAGCTCAACTTG | Yes |
| 822-1041 | Gi2464F  cggatccCAAGAGAACACATGTGCAAGC  Gi3123R  cagcggccgctaGGCTTCAAGTAGCTCAACTTG | Yes |
| 840-1041 | Gi2518F  cggatccTCAAGGACTGAAATGAATCC  Lynn/Gi3123R  cagcggccgctaGGCTTCAAGTAGCTCAACTTG | No |
| 1034-1173 End | Lynn/Gi3100F  cggatccCCTCAACTTGAGCTACTTGA  Lynn/GitermNot32a2  gtgcggccgctattgggacaaggatatagt | No |
| 789-893 | Lynn/GI-CT5for (NcoI)  accatggcCAAAGTTGTTGCCTCCATTGTTGACA  Lynn/GI-CT5rev (XhoI)  TCTCGAGTTAACCACAATAGAACCCTGCGAGTCTAT | Yes |
| T825A | Lynn/GI-mut6f  CTGGAAACAAGAGAACgcaTGTGCAAGCACCAC  Lynn/GI-mut6r  GTGGTGCTTGCACATGCGTTCTCTTGTTTCCAG | Yes |
| T829A | Lynn/GI-mut11f  AACACATGTGCAAGCgccACATGCTTTGATACAGC  Lynn/GI-mut11r  GCTGTATCAAAGCATGTGGCGCTTGCACATGTGTT | No |
| T830A | Lynn/GI-mut12f  ACATGTGCAAGCACCgCATGCTTTGATACAGCG  Lynn/GI-mut12r  CGCTGTATCAAAGCATGCGGTGCTTGCACATGT | Yes |
| T837A | Lynn/GI-mut13f  GATACAGCGGTGgCATCCGCCTCAAGG  Lynn/GI-mut13r  CCTTGAGGCGGATGCCACCGCTGTATC | Yes |
| S838A | Lynn/GI-mut14f  CAGCGGTGACAgCCGCCTCAAGGAC  Lynn/GI-mut14r  GTCCTTGAGGCGGCTGTCACCGCTG | Yes |
| S840A | Lynn/GI-mut7f  GGTGACATCCGCCGCAAGGACTGAAATGAATCC  Lynn/GI-mut7r  GGATTCATTTCAGTCCTTGCGGCGGATGTCACC | Yes |
| T834A | Lynn/GI-mut9f  ACCACATGCTTTGATgCAGCGGTGACATCCG  Lynn/GI-mut9r  CGGATGTCACCGCTGCATCAAAGCATGTGGT | Yes |
| S828A | Lynn/GI-mut10f  ACAAGAGAACACATGTGCAGCCACCACATGCTTTGATACAG  Lynn/GI-mut10r  CTGTATCAAAGCATGTGGTGGCTGCACATGTGTTCTCTTGT | Yes |

789 – KVVASIVDKAEPLEAYLKNTPVQKDSVTCLNWKQENTCAS**T**TCFDTAVTSAS

RTEMNPRGNHKYARHSDEGSGRPSEKGIKDFLLDASDLANFLTADRLAGFYCG - 893

**Supplemental Figure 1** Sequence of GI fragment expressed in CT5. The amino acid numbering is based on GenBank: AAT80910.1. The underlined T corresponds to T829 in intact GI.


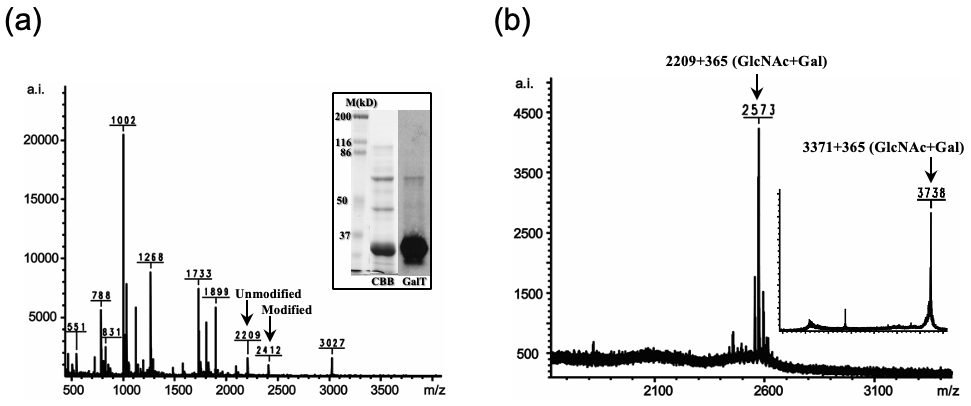


**Supplemental Figure 2** O-GlcNAc modified CT5 peptides were enriched by RCA I affinity chromatography. **(a)** MALDI-TOF analysis of trypsinized CT5 before capping with galactose and enrichment. Unmodified and modified QENTCASTTCFDTAVTSASR peptides with single O-GlcNAc (203 Da) were observed at *m/z* 2209 and 2412, respectively. The insert shows a protein blot demonstrating that CT5 co-expressed with SEC in *E. coli* was O-GlcNAc modified. The left two lanes show a portion of the stained gel with molecular weight markers followed by purified CT5 stained with Coomassie Brilliant blue (CBB). The blot (GalT) shows CT5 after labeling of GlcNAc residues with [^3^H] galactose. **(b)** Peptides produced with trypsin and enriched by RCA I column chromatography were analyzed using MALDI-TOF. CT5 QENTCASTTCFDTAVTSASR peptide (*m/z* 2209) containing single modification +365 Da (LacNAc) was observed at *m/z* 2573. The insert shows that QENTCASTTCFDTAVTSASRTEMNPRGNHK peptides (*m/z* 3371) containing a single modification (+365 Da) was observed at *m/z* 3736 after Lys-C digestion and enrichment.
